# Psychometric Characteristics of the Wish to Be Dead Scale (WDS) in Iranian Psychiatric Outpatients

**DOI:** 10.1007/s12144-016-9527-y

**Published:** 2016-11-21

**Authors:** Mahboubeh Dadfar, David Lester, Mohammad Kazem Atef Vahid

**Affiliations:** 1 Department of Clinical Psychology, School of Behavioral Sciences and Mental Health- Tehran Institute of Psychiatry, International Campus, Iran University of Medical Sciences, Tehran, Iran; 2 Psychology Program, Stockton University, Galloway, NJ USA

**Keywords:** Wish to be dead, Suicidal process, Psychiatric outpatients

## Abstract

The Wish to be Dead Scale (WDS) was administered to a convenience sample of 200 Iranian psychiatric outpatients. Using a Principal Component Analysis, two factors were identified, labeled Lack of purpose in life (F1), and Lack of interest in living (F2). The WDS had good reliability and significant positive correlations with scores on the Beck Suicide Ideation Scale and with other measures of mental ill-health. This study provides evidence of the usefulness of the WDS for assessing psychiatric patients.

## Introduction

According to the report of the World Health Organization (WHO) (2016), over 800, 000 people lose their lives due to suicide annually, and there are many more people who attempt suicide. Suicide is the second leading cause of death for those aged 15–29. 75% of global suicides occur in low- and middle-income countries. On average, a person dies by suicide every 40 s. There are cross-national differences in the patterns of suicide, including changes in the rates, characteristics and methods of suicide; and also the stigma and taboos regarding to suicide (WHO 2016, 2014).

As a result of the traditional culture in Iran, statistics about rates of suicide are inexact (Nazarzadeh, Bidel, Ayubi, Soori, & Sayehmiri 2013). Mosa Farkhany Khooban, EftekhariGol, and khosraviAsl (2012) found that estimated rates of suicide have increased by 60% in Iran in recent years. The average completed suicide rate was 10.95 per 100,000, and the mean age of the suicides was 29 years. Gorgi, Rezaeian, Rezaei, and Sheikh Fathollahi (2016) investigated the epidemiology of suicide and suicide attempts in Fars province of Iran from 2009 to 2012. The suicide rate was 3.85 per 100,000. The modal suicide was male (50.8%), single (49.7%), aged 15–24 years (42.9%), and a housewife (34.1%). The rate of suicide attempts was 99.53 per 100,000. The modal suicide attempter was female (60.5%), single (56.7%), and aged 15–24 years (57.3%). Rostami, Daliri, Sayehmiri, Delpisheh, and Sayehmiri (2016) used a meta-analysis to estimate the reported incidence of suicide attempts in Iran during 2001–2012: 91.65 per 100,000 overall, 82.2 for men and 115.79 for women.

The complexities of the causation of suicide make suicide prevention difficult. For the prevention of suicide, it is necessary that society coordinates its multiple sectors and encourages collaboration between the sectors, as well as developing a comprehensive and integrated approach (WHO 2016; US National Strategy for Suicide Prevention 2012). Regarding preventive suicide efforts the WHO has proposed a program called: The WHO Mental Health Action Plan 2013–2020.

There are many theories of suicide (Abramson, Alloy, Hogan, Whitehouse, et al. 2002; Beck, Steer, Kovacs, & Garrison 1985; Lester 1992), beginning with Durkheim’s classic theory (1897) over one hundred years ago. Shneidman (1992), the founder of the American Association of Suicide, developed a “cubic” model of suicide, proposing that the risk of suicidal behavior was determined by three factors - pain, perturbation, and press. He believed that unendurable psychological pain, derived from frustrated psychological needs, is a common stimulant in suicidal behavior.

An important recent psychological theory of suicide is Joiner’s (2005) Interpersonal Theory of Suicidal Behavior. This theory has stimulated a great deal of research in recent years (Lester, & Gunn 2012). Joiner’s theory proposes that two motivating elements and one acquired capability are present in those about to choose to die by suicide: the perception that one is a burden to others (perceived burdensomeness) and disruptions in one’s significant interpersonal relationships (thwarted belongingness). However, these two motivational elements are not sufficient in themselves. The potential suicide also has to overcome the fear and physical pain involved in suicide (the acquired capability for self-harm). This capability can be acquired by previous experiences of pain such as the experience of pain during physical and sexual abuse as a child, pain experienced as a result of accidents, physical diseases and surgery, and previous attempts at suicide. With an acquired capability for self-harm, people have no death fear and have a high pain threshold (Gunn, Lester, Haines, & Williams 2012). Lester and Gunn (2012) reported that thwarted *belongingness* was common in suicide notes, while perceived burdensomeness was less common. However, new research findings indicate the failure of the theory to fully explain all suicidal behavior (Anestis, & Joiner 2011; Bryan, Morrow, Anestis, & Joiner 2010; Van Orden, Witte, Cukrowicz, Braithwaite, Selby, & Joiner 2010). It seems that one of the main weaknesses of the theory is lack of attention to the role of emotions and process of emotion regulation (Hasani, & Miraghaie 2012).

There are three distinct important constructs in assessment of suicidal thinking and related behaviors: suicidal thoughts (active thoughts of killing oneself), *morbid* ruminations (thoughts about death, dying, and not wanting to be alive but with passive thoughts of killing oneself) and self-harm (underlying motivation is emotion regulation or for a reason other than death) (Simon, & Hales 2012; Rudd 2006).

Suicidal behavior has biological, social, psychological, and spiritual aspects. The identification of risk factors can help in the design of preventive interventions to reduce the incidence of suicide (Clarke, & Lester 2013; Ebrahimi, Kazemi, Fallahi Khoshknab, & Modabber 2014; Gunn & Lester 2012; Habibi, & Bagherian-Sararoudi 2013; Lester 2009, 2010; Lester, & Leenaars 2016). The causes of suicide include socio cultural, economic, interpersonal, psychiatric biological, and psychological (e.g., affective/emotional, and personality traits) factors (Hasani, & Miraghaie 2012). Psychological risk factors for suicide include an inability to regulate emotions, irrational thoughts and cognitive distortions, poor problem-solving skills, and attribution style (Tarsafi, Kalantar Kousheh, & Lester 2015).

Suicidal behaviors are the result of complicated interactions of medical, psychological, social and family factors, including age, gender, marital status, ethnicity, religion, culture, economic crisis, unemployment, living in metropolitan areas, climate, childhood trauma, psychiatric disorders and physical illnesses (Barth, Sogner, Gnambs, Kundi, Reiner, et al. 2011; Lester, & Gunn 2016a, 2016b; Szanto, & Waern, 2012). Nazarzadeh, Bidel, Ayubi, Soori, and Sayehmiri (2013) in a review of suicidal behavior in Iran found that family conflict was one of the most important causes of suicide attempts (30%) in the recent years, followed by marital problems (26%), economical stressors (12%), and educational failure (5%).

The most important psychological long-term risk factors for suicide include depression, psychiatric disorder, a history of hospitalization in a psychiatric hospital, stress in personal intimate relationships, a family history of suicide, a history of suicide attempts, social isolation, and alcoholism and drug addiction (Modabber, Ebrahimi, Kazemi, & Fallahi Khoshknab 2014).. Warning signs for suicide include impulsive and aggressive behaviors, direct or indirect talk about suicide, sudden changes in behavior, loss of loved ones, and unbearable hurt and indignation/rage (Lester, & Stack 2015; Tartaro, & Lester 2015). May, Klonsky, and Klein (2012) identified six significant predictors of future suicide attempts: cluster A personality disorder, cluster B personality disorder, lifetime substance abuse, baseline anxiety disorder, a poor maternal relationship, and poor social adjustment. They reported that comorbid cluster B personality disorder emerged in a multiple regression analysis as the only robust predictor of future suicide attempts among depressed suicide ideators. Hakim Shooshtari and Khanipour (2014) reported that the severity of self-harm predicted future attempts at suicide, and that common risk factors for future attempted suicide included depressive symptoms, posttraumatic stress disorder and borderline personality disorder, difficulties in regulating emotions and a reduction of fear of death predicted future suicidal behavior in Iranian adolescents who had engaged in self-harm. Ebrahimi, et al. (2014) found that there was a significant relationship between suicide ideation and gender, occupation, education, physical disorders, and the use of non-psychotropic drugs among Iranian depressed patients who have suicidal ideation.

Suicidal ideation is one of the most important risk factors for suicide (Joiner, Pettit, Walker, Voelz, et al. 2002; Groleger, Tomori, & Kocmur 2003) and is a more powerful predictor of suicide than other variables (Witte, Fitzpatrick, Joiner, & Schmidt 2005). Therefore, it is important to identify the variables that predict the transition from suicidal thoughts to attempted and completed suicide (Haerian, Salmasian, & Friedman 2012; Krysinska, Lester, Lyke, & Corveleyn 2015; May, Klonky, & Klein 2012). It is estimated that each suicide attempt increases risk for successful suicide to 32% (Baca-Garcia, Perez-Rodriguez, Oquendo, Keyes, Hasin, et al. 2011). In agreement with this, Khajehmougahi, Behrouzian, and Ghanavati (2010) found that one of the most significant factors in prediction of suicide in Iran was a previous attempted suicide.

Cognitive variables also play a role in understanding suicidal behavior. Hasani and Miraghaie (2012) found that self-blame, rumination and catastrophizing correlated positively with suicidal ideation. Hopelessness is a strong predictor of suicidal behavior (Beck, Steer, Beck, & Newman 1993; Beck, Brown, Berchick, Stewart, & Steer 1990; Beck, et al. 1985), along with feelings of defeat and entrapment and falling short of internalized standards (Lester 1998; Lester, & Leenaars 2016). Rudd, Joiner and Rajab (2001) reported that hopelessness is a better predictor of suicidal behavior than is depression. The suicidal thoughts and tendencies in suicide attempters, and after suppression of despair, desire to die is reduced. On the Beck Hopelessness Scale (BHS), Khajehmougahi, et al. (2010) found that hopelessness and male gender were the best predictors of attempting suicide in Iranian psychiatric patients.

The question arises of whether there are additional variables that would improve the prediction of future suicidal behavior, especially in the short-term. Marušic, Roškar, Svetii, and Zorko (2012) proposed the psychological construct of a desire to be dead and argued that this desire is an initial step in the process leading to suicide. Marušic, et al. proposed a sequence of having a desire to be dead, thinking about suicide as an option, followed by suicidal actions (attempted and completed suicide). The wish to be dead is one of motivations for suicidal attempts. Having a strong wish to be dead most likely is accompanied by feelings of depression, and those who have a strong wish to be dead typically have ruminated about this idea for many years. Those who wish to be dead may well have failed to take care of themselves when faced with illnesses, and may even have engaged in risky behaviors which, in turn, may have resulted in injury and pain. Such individuals may experience a reduced fear when contemplating self-harm and suicide and may be more willing to endure the anticipated pain. Therefore, it would be of potential clinical importance to be able to assess the wish to be dead in psychiatric patients in order to provide additional warnings signs for suicide. Lester (2013) developed a scale to measure this wish to be dead (the Wish to be Dead Scale: WDS) in order to improve the assessment and prediction of suicide risk in clinical practice.

Nations differ in culture, language, and historical events. Despite the good reliability and validity of the WDS in American college students (Lester 2013), and Iranian college students (Dadfar, Lester, Atef Vahid, and Abdel-Khalek 2016c), there are no published studies using clinical samples. The WDS could be useful in research and in clinical practice. It is important, therefore, to validate the WDS in psychiatric patients (rather than relying on the use of non-clinical samples of college students). The aim of the present study was to evaluate the psychometric characteristics of the WDS, that is, its reliability, validity and factorial structure, in Iranian psychiatric outpatients. In addition, the research sought to identify other psychological variables that are associated with the wish to be dead.

## Methods

### Participants

A convenience sample of 200 Iranian volunteer psychiatric outpatients was selected from the psychiatric and psychological clinics at the School of Behavioral Sciences and Mental Health-Tehran Institute of Psychiatry in the Iran University of Medical Sciences. The mean age of the patients was 34.0 years (SD = 13.6); the mean duration of their mental disorder was 7.7 years (SD = 7.9); 72.5% were female; 50.0% were single; 41.5% were married and 6.5% divorced; 38.5% had children; 28% were housewives and 23.5% were employed; 19% had a depressive disorder and 11.5% an anxiety disorder; 46.5% had family history of mental disorders; 29% had physical disorders; 38.5% received treatment (40% drug therapy, 4% psychological therapy, 33.5% both, and 22.5% missing data); 34.5% had a history of suicidal ideation, 22.5% had a history of suicidal attempt, 8.5% had current suicidal thoughts, and 4.5% had made a recent suicidal attempt.

### Procedure

The purpose of the study was explained to the patients. The confidentiality of the patients’ information was maintained, they were told that their participation in the study was voluntary, and the patients completed consent forms. A Farsi version of the Wish to be Dead Scale (WDS) was administered to the sample in order to assess the reliability, validity and factorial structure of the scale. The time period for the test-retest analysis was one-week**.**

We identified psychological variables that might be correlated with the wish to be dead, including the Beck Suicide Ideation Scale (BSIS), the Beck Hopelessness Scale (BHS), the Beck Depression Inventory, short version (BDI-13), the Death Obsession Scale (DOS), the Kessler Psychological Distress Scales (K10/K6), the General Health Questionnaire-12 (GHQ-12), the Loneliness Scale (LS), Passive Death Wishes (one question), Current Suicidal Thoughts (one question), and Current Preference for Death (one question). Our rationale for each measure’s inclusion in this study, especially in this sample, was based on previous research on risk factors and predictive factors for completed suicide, including suicidal ideation, hopelessness, depression, obsession with death, general psychological distress, general health, and loneliness. The patients were administered the scales individually. There were no missing data. All patients completed the scales fully.

### Measures

1. The Wish to be Dead Scale (WDS: Lester 2013) has 10 items and two Likert and true/false answer formats. The WDS had moderately good test-retest reliability and internal consistency in a sample of American undergraduate students enrolled in psychology courses. Cronbach alpha was 0.82, and the two-week test-retest correlation was 0.87 (Lester 2013). Dadfar, Lester, Atef Vahid, and Abdel-Khalek (2016c) validated a Farsi version of the WDS using a sample of Iranian college students. They found to have good internal reliability (Cronbach alpha 0.73) and one and two-week test-retest reliabilities (0.77 and 0.72, respectively), and good construct validity with other psychological measures in a sample of Iranian university students (Atef Vahid, Dadfar, Abdel-Khalek, & Lester 2016). A typical item is, “I have occasionally fantasized about my funeral.”
2. The Beck Suicide Ideation Scale (BSIS) consists of 19 items and has shown good reliability and validity in American and Turkish samples (Ozdel, Varma, Atesci, Oguzhanoglu, et al. 2009; Auerbach, Millner, Stewart, & Esposito 2015). There was a negative correlation between BSIS scores and scores on a new version of the Reasons for Living Inventory for Adolescents (RFL–A) in Iranian adolescents (*r* = −0.48 *p* < .05: Khodabakhshi Koolaee, Mahmmodi, & Ozuoni Davaji 2008). Cronbach alpha for the Farsi version of the BSIS was 0.90 in Iranian high-school students (Sajadi, Hajjari, Zardar, et al. 2014) and 0.93 in a sample of Iranian psychiatric patients (Dadfar & Kalibatseva 2016). Items are answered using a Likert-type format: 0 (moderate to strong), 1 (weak), 2 (none). A typical item is, “I have the courage to commit suicide.”
3. The Beck Hopelessness Scale (BHS) is a 20-item self-report inventory, answered using a 5-point Likert-type format, deigned to measure key aspects of hopelessness such as feelings about the future, loss of motivation, and expectations (Beck, Weismann, Lester, & Trexler 1974). The BHS has been shown to be a reliable and valid measure in 40 years of use (Perczel Forintos, & Sallai 2010). Cronbach alpha for a sample of Iranian psychiatric patients was 0.64 (Dadfar & Kalibatseva 2016). There was a negative correlation between BHS and the RFL–A in Iranian adolescents (*r* = −0.61 *p* < .05: Khodabakhshi Koolaee, Mahmmodi, & Ozuoni Davaji 2008). A typical item is, “I never get what I want so it’s foolish to want anything.”
4. The Beck Depression Inventory, short version (BDI-13) measures the severity of depression symptoms**.** The Farsi version of the BDI-13 was developed by Hojat, Shapurian, and Mehryar (1986). The BDI-13 has 13 items answered on a 4-point Likert-type scale. It has proved to have good reliability and validity (Al-Yasiri, & AbdKarkosh 2013). Cronbach alpha for a sample of Iranian psychiatric patients was 0.85 (Dadfar & Kalibatseva 2016). A typical item is, “I am so sad or unhappy that I can’t stand it.”
5. The Death Obsession Scale (DOS, Abdel-Khalek 1998) has 15 items answered using a 5-point rating scale: 1: No, 2: A little, 3: A fair amount, 4: Much, and 5: Very much. Total scores can range from 15 to 75. The DOS had high internal consistency (Cronbach’s α = 0.90) and high test-retest reliability over a one-week period (*r* = 0.91; Abdel-Khalek 1998). Scores on the scale were associated with scores for general obsessiveness and general anxiety and depression (Pearson *r’*s ranging from 0.34 to 0.45), indicating reasonable construct validity (Abdel-Khalek 1998). Cronbach alpha for a sample of Iranian psychiatric patients was 0.94 (Dadfar & Kalibatseva 2016). A typical item is: “I think about death continuously.” The scale has been translated into Persian (Mohammadzadeh, Asgharnejad Farid, & Ashouri 2009) and used in several studies (e.g., Rajabi, 2007; Moripe, & Mashegoane 2013; Dadfar, & Lester 2015) with good reliability and construct validity.
6. The Kessler Psychological Distress Scale-10 (K10; Kessler et al. 2003) consists of 10 items that screen for mental illness in the general population, scored on a 5-point scale: none of the time (0); a little of the time (1); some of the time (2); most of the time (3), and all of the time (4). A typical item is, “During the last 30 days, about how often did you feel nervous?” Kessler, et al. (2003) reported strong correlations with other similar measures and good predictive ability for a diagnosis of psychiatric disorder. Cronbach alphas for the Farsi version of the K10 were 0.88 in college students (Atef Vahid, Dadfar, Kessler, Bahrami, & Lester 2015), 0.92 in psychiatric outpatients (Dadfar, Lester, Atef Vahid, & Nasr Esfahani 2016a), and 0.80 in women with breast cancer (Vaziri, & Lotfi Kashani 2011; Lotfi Kashani, Vaziri, Zeinolabedini, & Zeinolabedini 2014). Cronbach alpha for a sample of Iranian psychiatric patients was 0.94 (Dadfar & Kalibatseva, 2016). The Kessler Psychological Distress Scale-6 (K6) is a short version of the K10. It has good validity and reliability (Andersen, Grimsrud, Myer, Williams, Stein, & Seedat 2011; Mitchell, & Beals 2011; Krynen, Osborne, Duck, Houkamau, & Sibley 2013; Suraweera, Hanwella, Sivayokan, & Varuni de Silva 2013; Yiengprugsawan, Kelly, & Tawatsupa 2014; Al-Hamzawi, Bruffaerts, Bromet, AlKhafaji, & Kessler 2015; Dadfar, Atef Vahid, Lester, & Bahrami 2016b).
7. The General Health Questionnaire-12 (GHQ-12) has been used as a mental health screening tool in primary care, general medical practice and community surveys to detect minor and non-psychotic psychiatric conditions. The GHQ-12 is a quick, screening tool, with 12 items, answered on a 4-point Likert-type scale. It has good reliability (Cronbach alpha was 0.87 in Iranian young people (Montazeri, Harirchi Shariati, et al. 2003) and good validity. Cronbach alpha for a sample of Iranian psychiatric patients was 0.76 (Dadfar & Kalibatseva 2016). The GHQ-12 has been used in many countries (Mann, Paglia-Boak, Adlaf, et al. 2011; Yaghubi, Karimi, Omidi, et al. 2012; Dadfar, Lester, Atef Vahid, et al. 2015; Tagharrobi, Sharifi, & Sooky 2014-2015). A typical item is, “Thinking of self as worthless.”
8. Loneliness was evaluated with the question, “How frequently have you felt lonely over the past week?” This item is from the Center for Epidemiological Studies of Depression questionnaire (CES-D) (Radloff 1977; Shiovitz-Ezra, & Ayalon 2010; Ayalon, & Shiovitz-Ezra 2011). Response options are: not at all (1), part of the time (2), most of the time (3), and almost all the time (4), with a higher score indicating greater loneliness (Shiovitz-Ezra, & Ayalon 2010).
9. Passive Death Wishes were evaluated with the question,“In the past month, have you felt that you would rather be dead?” This item comes from the EURO-D, a face-to-face administered measure of depression (Prince, Reischies, Beekman et al. 1999; Ayalon, & Shiovitz-Ezra 2011). Response options are: “yes” (1) participant has mentioned passive death wishes versus “no” (0) no thoughts mentioned (Ayalon 2010; Raue, Morales, Post, et al. 2010).
10. Current Suicidal Thoughts were evaluated with a question written by the present authors: “At the present time, do you have suicidal thoughts?”. Response options were: “yes” (1) participant has mentioned current suicidal thoughts versus “no” (0) no thoughts mentioned.
11. Current Preference for Death was evaluated with the question written by the present authors: “At the present time, would you prefer to be dead?” Response options were: “yes” (1) participant has mentioned current suicidal thoughts versus “no” (0) no thoughts mentioned.

### Analysis

The data were analyzed using Means, Standard Deviations, Pearson Correlations, Cronbach Alpha coefficients, the Kaiser-Meyer-Olkin Measure of Sampling Adequacy (KMO) and Bartlett’s Test of Sphericity for justifying the use of the factor analysis, and Chi-Square statistics. The extraction and rotation methods were Principal Component Analysis and Varimax rotation with Kaiser Normalization, respectively. The factor analysis explores the most appropriate and relevant psychological inventory scores with the highest weights on the factors identified. In order to assess the accordance and labeling the extracted factors, Varimax rotations provide orthogonal (independent) factors while Promax rotations permit non-orthogonal (oblique) factors). In general, Varimax rotations increase the likelihood that each score is loaded on only one factor, and so was chosen for the present analysis. We used SPSS/WIN 16.0 program for analyzing the data.

The mean scores, Cronbach alphas and correlations with WDS scores for the present sample are shown in Table 1.

**Table 1:** Means, SDs and Cronbach alphas for the scales

Scales	Mean	SD	Numbers of items	Answer range (Likert or False-True)	Cronbach alpha	Pearson r with WDS
Beck Suicide Ideation Scale (BSIS)	2.78	5.54	19	0–2	0.93	0.44**
Beck Hopelessness Scale (BHS)	10. 57	2.00	20	False (0) -True (1)	0.64	0.21
Beck Depression Inventory (BDI)	11.03	7.55	13	0–3	0.86	0.69**
Death Obsession Scale (DOS)	27.78	13.68	15	1–5	0.94	0.40**
Kessler Psychological Distress Scale (K10)	17.00	9.89	10	0–4	0.94	0.63**
Kessler Psychological Distress Scale (K6)	11.64	5.90	6	0–4	0.89	0.46**
General Health Questionnaire (GHQ-12)	18.00	7.94	12	0–3	0.76	0.55**
Loneliness Scale (LS)	0.76	0.42	1	1–4	0.87	0.37**
Passive Death Wishes	1.66	0.47	1	False (0) -True (1)		- 0.57**
Current Suicidal Thoughts	1.92	0.26	1	False (0) -True (1)		- 0.35*
Current Preference for Death	1.76	0.42	1	False (0) -True (1)		- 0.43**

*two-tailed *p* < .05, **two-tailed *p* < .01

## Results

### Reliability

The Cronbach alpha for the WDS was 0.82, the split-half reliability 0.55, the Spearman-Brown coefficient 0.71, and the one-week test-retest reliability 0.95, indicating good reliability.

The inter-correlations between the items (see Table 2) ranged from 0.07 to 0.76, and the item-total correlations ranged from 0.40 to 0.75 with a median of 0.65.

**Table 2:** Correlations between items and with the total score of the Wish to be Dead Scale1

Item	1	2	3	4	5	6	7	8	9	10	Total
1	1										
2	.333**	1									
3	.184**	.495**	1								
4	.085	.309**	.482**	1							
5	.220**	.459**	.386**	.161*	1						
6	.131	.189**	.174*	.242**	.210**	1					
7	.239**	.356**	.374**	.187**	.468**	.251**	1				
8	.301**	.265**	.344**	.110	.340**	.261**	.495**	1			
9	.343**	.358**	.283**	.075	.327**	.270**	.399**	.588**	1		
10	.373**	.400**	.303**	.028	.361**	.270**	.448**	.636**	.760**	1	
Total	.516**	.665**	.637**	.402**	.638**	.480**	.680**	.711**	.724**	.753**	1

**significant at the 0.01 level, *significant at the 0.05 level

1 We used the correlations (>.40) n bold entries to more clearly correlate items and with total score

### Factor Analysis

The criteria for a factor analysis were evaluated using Kaiser-Meyer-Olkin Measure of Sampling Adequacy (KMO) and Bartlett’s Test of Sphericity. The KMO was 0.83, indicating the adequacy of the sample, and Bartlett’s Test of Sphericity (X^2^ = 672.30, df = 45, *p* < .001) indicated that the factor analysis was justified. To investigate the factor structure of the scale, a Principal Component Analysis with a Varimax rotation and Kaiser Normalization were used.^1^

Two components with eigenvalues greater than one were extracted (see Table 3). Factor 1 (4 items) can be labeled “Lack of purpose and usefulness in life” and included items such as “I sometimes think that there is no purpose to life.” Factor 2 (3 items) can be labeled “Lack of interest in living” and included items such as “I sometimes think that death would solve my problems.”

**Table 3:** Factor loadings (>0.5) of the Wish to be Dead Scale in 200 Iranian psychiatric outpatients1

Wish to be Dead Scale (WDS) Items	Component		% answering true
	1	2	
1. There have been times when I wished that I were dead.	**.51**	.14	71.0%
2. I sometimes think that death would solve my problems.	.36	**.63**	31.0%
3. Sometimes I wish I could go to sleep for several years.	.21	**.78**	30.5%
4. I occasionally day-dream about being dead.	-.13	**.81**	19.0%
5. I sometimes think that there is little point in living.	.43	.49	60.0%
6. I have occasionally fantasized about my funeral.	.30	.32	32.0%
7. I have on occasions lost my desire to live.	**.56**	.40	69.0%
8. I sometimes wish that I had never been born.	**.77**	.16	54.5%
9. I sometimes think that there is no purpose to life.	**.84**	.10	47.5%
10. It occasionally crosses my mind that life is not worth living	**.88**	.10	44.5%
Eigenvalue	3.99	1.41	
% of Variance	39.91	14.13	

Factor 1 (items: 1, 7, 9, and 10): Lack of purpose and usefulness in the life. Factor 2 (items: 2, 3, and 4): Lack of interest to live

1 We used the high loadings (>.50) ) in bold entries to more clearly differentiate the factors

### Construct Validity

The correlations between WDS scores and the scale scores (see Table 1) were: BSIS *r* = 0.44 (*p* < .01), BDI *r* = −0.69 (*p* < .01), BHS *r* = 0.21 (ns), DOS *r* = 0.40 (*p* < .01), K10 *r* = 0.63 (*p* < .01), K6 *r* = 0.46 (*p* < .01), GHQ-12 *r* = 0.55 (*p* < .01), LS *r* = 0.37 (*p* < .01), Passive Death Wishes *r* = −0.57 (*p* < .01), Current Suicidal Thoughts *r* = −0.33 (*p* < .05), and Current Preference for Death *r* = − 0.43 (*p* < .01), all in the direction to be expected, thereby indicating good construct validity.

### A Test of the Theory

Scores on the Beck Suicidal Ideation Scale (BSIS) were significantly associated with both scores on the WDS (*r* = 0.44, two-tailed *p* < .01) and the Beck Depression Inventory (BDI: *r* = 0.46, *p* < .01), but not with scores on the hopeless scale (BHS: *r* = 0.04). Since the association between BDI and WDS scores was only 0.69, it appears that the WDS and BDI might together provide a better predictor of current suicidal ideation than either alone.

## Discussion

The aim of the present study was to explore the validity and reliability of Lester’s (2013) Wish to be Dead Scale (WDS) in psychiatric patients, the first study of the WDS in such patients. The WDS had good internal consistency and one-week test-retest reliability in this sample of Iranian psychiatric outpatients.

In a factor analysis, the present study identified two components of the WDS: (i) lack of purpose and usefulness in the life, and (ii) lack of interest in living. These results inconsistent with those using samples of university students reported by Lester (2013) who found only a single factor and Dadfar, et al. (2016c) who found three factors (labeled lack of purpose and usefulness in life, lack of interest in living, and fantasizing about being dead). The number of factors identified in a sample may depend on the degree to which the members of the sample have thought about suicide. So, for example, psychiatric patients may have had more personal experience with suicidal ideation and behavior as compared to college students in general, and so have more complex thoughts surrounding the wish to be dead. Further research should study the WDS in psychiatric patients who are or who have been suicidal and those who are non-suicidal. Nevertheless, the wish to be dead may be comprised of several components, and it would be of interest to assess their correlates separately in future studies.

The only Structural Equation Modeling (SEM) that might apply to the data is a confirmatory factor analysis (and not path analysis, LISREL, or latent growth modeling). However, we are exploring the structure and not confirming any hypothesized structure or a structure that we had identified in previous research. In previous research on college students, we and others have used principal component analyses. We used a Varimax rotation because it simplifies the factor matrix and maximizes the sum of the variances, and more clearly differentiating the factors.

The means score of the sample on the WDS was 4.59 (SD = 2.94), and three items were agreed with by more than half of the sample: There have been times when I wished that I were dead (71.0%), I have on occasions lost my desire to live (69.0%), and I sometimes think that there is little point in living (60.0%). It appears, therefore, that elements of the wish to be dead are common in this sample of psychiatric patients. In Dadfar, et al.’s study of college students (Dadfar, et al. 2016c), the two items most frequently agreed with were: There have been times when I wished that I were dead, and I have on occasions lost my desire to live, both among the most common in the present sample of psychiatric patients.

Scores on the WDS were associated with scores on measures of suicide ideation, depression, death obsession, general psychological distress, general health, and loneliness. These associations provide evidence for the construct validity of the WDS in psychiatric patients. There was also a weaker (non-significant) association with hopelessness. Therefore, the WDS may be useful for assessing preliminary stages in the pathway to suicide and for estimating the risk for future suicidal behavior in psychiatric outpatients, in addition to the established risk factors for suicide (depression, anxiety, and personality disorders, especially cluster B [i.e., borderline personality disorder].

The present study had some limitations. The sample was from a convenience clinical population of psychiatric outpatients. Future research should explore the WDS in psychiatric inpatients and in patients with differing psychiatric diagnoses. A large number of scales were used to examine the convergent and discriminant validity of the WDS, and the results may have been less supportive had other scales been used. It would useful in future research to base the choice of scales on a theoretical model of the suicidal process. For example, the WDS could be used with measures of thwarted belongingness, perceived burdensomeness and the acquired capability for self-harm to see if the WDS adds to the predictive power of the battery of tests for suicidal behavior.

Regarding testing the ability of the WDS to predict suicidal behavior, we have only past suicidal behavior for the patients, and Marusic’s theory proposes that the wish for death is an early stage in the suicidal process. Future research should explore the ability of the WDS (alone and in combination with other inventories such as the BDI) to predict *future* suicidal behavior. It is hoped, therefore, that this study will stimulate further research on the WDS in clinical practice, especially with psychiatric inpatients, attempted suicides, substance abusers, and those with cluster A and B personality disorders.

## Footnotes

1 We used the Varimax rotation because the factors obtained were better differentiated, making interpretation easier. We also used high loadings (> .50) to more clearly differentiate the factors.
